# The wavelength of the incident light determines the primary charge separation pathway in Photosystem II

**DOI:** 10.1038/s41598-018-21101-w

**Published:** 2018-02-12

**Authors:** Andrea Pavlou, Julien Jacques, Nigar Ahmadova, Fikret Mamedov, Stenbjörn Styring

**Affiliations:** Molecular Biomimetics, Department of Chemistry — Ångström, Box 523, Uppsala University, SE 751 20 Uppsala, Sweden

## Abstract

Charge separation is a key component of the reactions cascade of photosynthesis, by which solar energy is converted to chemical energy. From this photochemical reaction, two radicals of opposite charge are formed, a highly reducing anion and a highly oxidising cation. We have previously proposed that the cation after far-red light excitation is located on a component different from P_D1_, which is the location of the primary electron hole after visible light excitation. Here, we attempt to provide further insight into the location of the primary charge separation upon far-red light excitation of PS II, using the EPR signal of the spin polarized ^3^P_680_ as a probe. We demonstrate that, under far-red light illumination, the spin polarized ^3^P_680_ is not formed, despite the primary charge separation still occurring at these conditions. We propose that this is because under far-red light excitation, the primary electron hole is localized on Chl_D1_, rather than on P_D1_. The fact that identical samples have demonstrated charge separation upon both far-red and visible light excitation supports our hypothesis that two pathways for primary charge separation exist in parallel in PS II reaction centres. These pathways are excited and activated dependent of the wavelength applied.

## Introduction

Several billion years ago, the evolution of photosynthetic organisms capable of splitting water into molecular oxygen (O_2_), protons [H^+^], and electrons (*e*^−^) with the mere absorption of sunlight, completely altered the course of evolution of life^1,2^. The complex photosynthetic reactions in these organisms (plants, algae and cyanobacteria), allow them to take in carbon dioxide (CO_2_), water and light energy to produce carbohydrates and O_2_, vital products for life sustainment. These light-dependent reactions take place in two multi-protein membrane complexes known as Photosystem II (PS II)^3^ and Photosystem I (PS I)^4^. PSII absorbs photons to use their energy to oxidize water, thereby supplying PS I with electrons to be used in carbon fixation^5,6^.

In PS II, absorption of a photon first results in excitation of photosynthetic pigments and very rapidly thereafter in the primary charge separation reaction between the primary donor of PS II, commonly referred to as P_680_, and the first electron acceptor, pheophytin (Pheo). In a series of subsequent electron transfer reactions, electrons are transferred from water (that is split into O_2_ when oxidized) to the first and second quinone electron acceptors, Q_A_ and Q_B_^7,8^. Unlike anoxygenic phototrophic bacteria, which contain bacteriochlorophylls that can drive anoxygenic photosynthesis at wavelengths longer than 900 nm, oxygenic photosynthesis was thought to be limited to wavelengths below *ca* 690 nm, also known as the red drop phenomenon as defined by^9^. The reason behind this difference was attributed to the high energy required for water oxidation in oxygenic photosynthesis, where O_2_ is released as a by-product. Lower energy photons above *ca* 690 nm were assumed to be unable to generate a redox potential that is high enough to drive water oxidation, while concomitantly retaining a negative excited state redox potential on the acceptor side required for the reduction of the primary electron acceptor^10^.

The notion that oxygenic photosynthesis can only be driven by photons with a maximum absorbance at or below ~690 nm involving Chl *a*, drastically changed with the discovery of the cyanobacterium *Acaryochloris marina*^11–13^. This photosynthetic cyanobacterium has ~95% of its Chl *a* substituted by Chl *d*, which is red-shifted with an absorbance maximum of ~710 nm. Despite this, *A. marina* is still able to oxidize water^14,15^. This results in efficient photochemistry taking place in a submarine ecosystem deficient in visible light, but largely penetrated by far-red light^16–18^. Thus, it is clear that photosynthetic water oxidation in nature can occur also with photons of energy lower than 690 nm. Interestingly, this also holds for PS II in green algae^19^ and higher plants^20,21^ containing a Chl *a/b* antennae without any Chl *d*. Spectroscopic investigation of the different electron transfer reactions in PS II, defined the far-red limit for water-oxidizing photochemistry in PS II to 780 nm (single flash) up to 800 nm (photoaccumulation)^22^. Based on these findings, an alternative charge separation pathway was proposed to occur upon far-red light excitation. When this pathway is activated, a state denoted X* that is lower in energy compared to P_680_* (formed in visible light) is formed in PS II. Despite its lower energy, X* is able to trigger the charge separation, reduce Pheo and oxidize tyrosine Z (Y_Z_).

This is an important evolutionary finding as it has redefined the required threshold energy for oxygenic photosynthesis, since X^*^ apparently works with an energetic threshold which is lower than P_680_*^22^. The existence of such a state was suggested previously, but never shown to induce charge separation at room temperature or at wavelengths as high as 800 nm. Although oxygen evolution and variable chl *a* fluorescence have been observed in sunflower (*Helianthus annuus*) and bean (*Phaseolus vulgaris*) leaves using wavelengths up to 780 nm^21^, the maximum long wavelength with which successful charge separation was achieved was 730 nm, as demonstrated by spectral hole-burning experiments on PS II core complexes at cryogenic temperatures (1.5 K)^23,24^. This range has now been extended to *ca* 780–800 nm where complete water oxidation can be achieved at room temperature in different PS II preparations^22^.

Mokvist *et al*., 2014 provided further insight into the electron transfer reactions in PS II, induced by far-red photochemistry^25^. It was shown that at 5 K, Y_Z_ was the preferred secondary electron donor in far-red light. This is different from the situation with visible light where the Cyt *b*_559_/Chl_Z_/Car_D2_ secondary pathway was equally important^25^.

**Figure 1:**
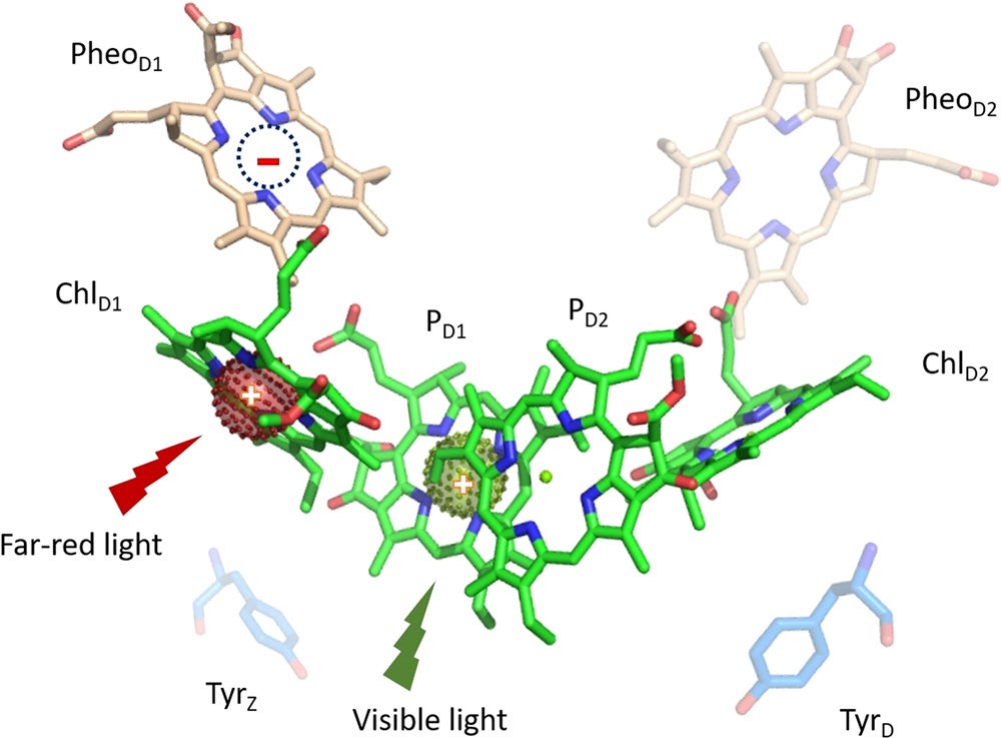
Structural representation of the redox active components in the PS II reaction center.

This observation led to the proposal of a different first stable charge pair denoted as P_x_^+^ Q_A_^−^ being formed under far-red light, as compared to the normal P_D1_^+^ Q_A_^−^ under visible light excitation. The proposed electron hole in P_x_^+^ was suggested to be residing on the Chl_D1_ molecule in the PS II reaction centre at 5 K. The observation also further supported the proposed existence of a low-energy threshold charge separation pathway, where the primary donor is Chl_D1_ rather than P_D1_^25^. (See Fig. 1 which shows the redox active components in the PS II reaction centre from ref.^26^).

In this study, we have used the spin polarized triplet EPR signal from ^3^P_680_, to further probe the photochemistry following visible or far-red illumination. Our results strengthen the hypothesis that the stable cation formed by the charge separation resides at Chl_D1_ when the excitation energy is provided by long wavelength (far-red) light, rather than on P_D1_ of the P_D1_/ P_D2_ Chl pair, which is the normal situation in visible light excitation.

## Results

### Functionality of over-reduced PS II

The experimental protocol to achieve reliable and quantitative double reduction of Q_A_ in PS II enriched membranes of our type (so called BBY particles), is complicated and has several potential pitfalls. The double reduction protocol involves reduction with sodium dithionite using benzyl viologen as mediator for several hours and demands exclusive handling under anaerobic and dark conditions^27–29^. Also the re-oxidation protocol is difficult and could potentially lead to sample damage^27^. It was therefore important to test for the integrity of the critical electron transfer reactions both after double reduction and after the re-oxidation of the samples used here.

#### Status of the charge separation reaction

The most important reaction in the current study is the primary charge separation reaction. In PS II centres with Q_A_H_2_ (double reduced and protonated Q_A_) or samples lacking Q_A_, the primary charge separation can be followed by photo-accumulation of the Pheo^−^ radical under reducing conditions^30–32^. This reaction is also known to be functional using far-red illumination^22^.

**Figure 2:**
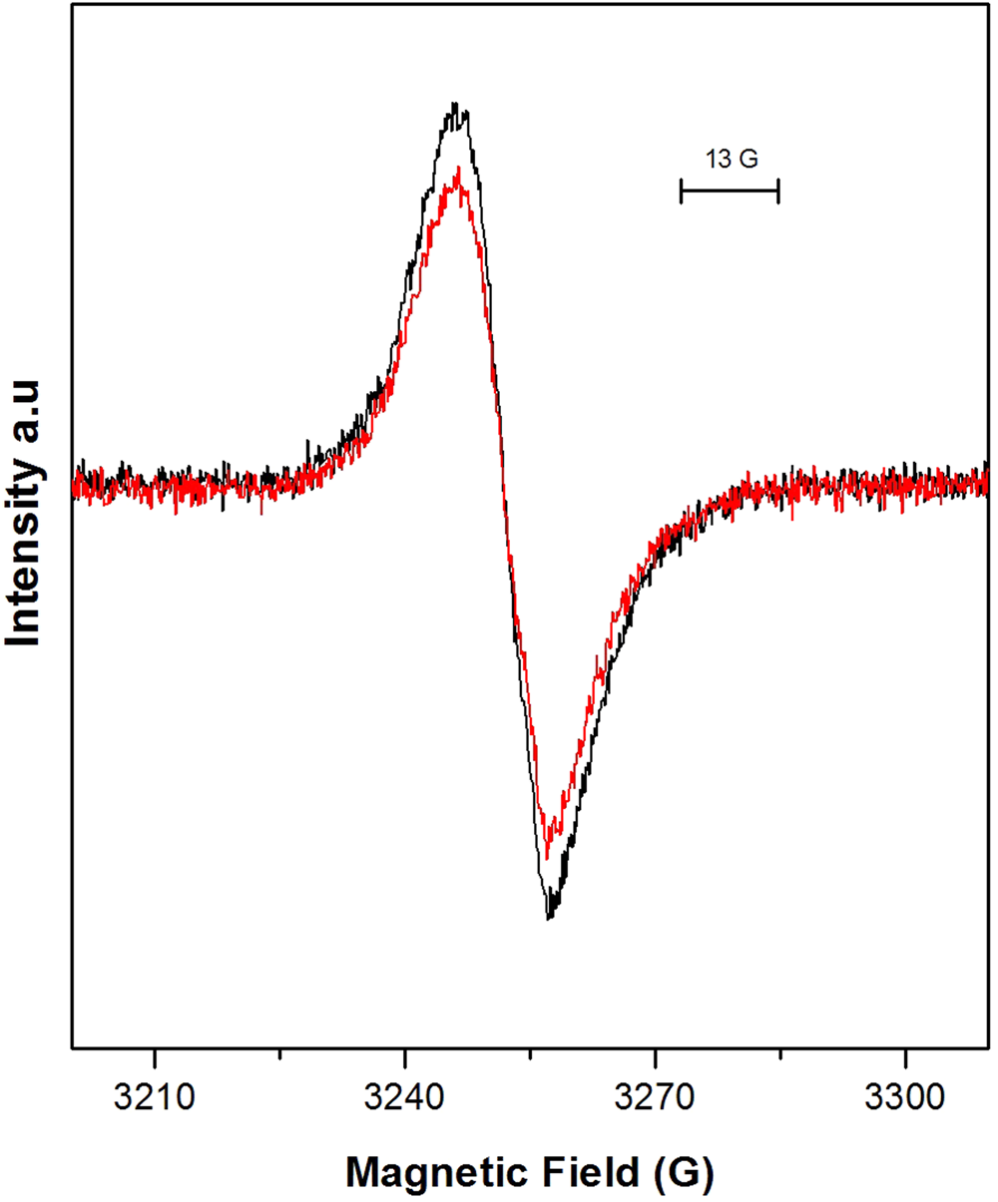
EPR signal of the Pheo^−^ radical in doubly reduced PS II membranes, after continuous illumination with white (black) and far-red at 732 nm light (red), at 20 °C, for 6 and 10  min respectively. EPR conditions were as follows: microwave power 1.27 μW, microwave frequency 9.139 GHz, modulation amplitude 3.5 G, temperature, 15 K.

Figure 2 shows the EPR signal from the Pheo^−^ radical in PS II enriched membranes where Q_A_ was doubly reduced. The radical EPR spectrum formed by either visible or far-red (732 nm) light was 13G wide with *g* = 2.0035^30–32^. Both parameters are indicative that the signal originates from the Pheo^−^ radical. Thus, the mere formation of the signal is an indication that PS II was able to perform the charge separation reaction despite being exposed to the dithionite/benzyl viologen reduction protocol. The size of the EPR spectrum from Pheo^−^ can be compared to the size of the EPR spectrum from the Y_D_^•^ radical in the corresponding intact PSII sample (non-reduced) that amounts to 1 radical/PSII reaction center^33,34^. In this particular experiment, exposure to white light resulted in Pheo^−^ radical formation in 70% of PS II, while the illumination at 732 nm resulted in Pheo^−^ radical formation in 55% of PS II. We can thus conclude that ≥70% of the PS II centres can perform the primary charge separation after the reduction treatment. In addition, it seems that 732 nm is nearly as efficient as white light, the small difference probably reflecting the weaker light source used at 732 nm.

#### Status of the Mn-cluster

It is generally thought that extensive reduction of PS II results in inactivation of the *OEC* through removal of the CaMn_4_—cluster. This also holds for our samples that showed typical characteristics of samples lacking the CaMn_4_-cluster. After the re-oxidation procedure, no O_2_ evolution could be detected. Corroborating this, neither the Split S_1_ EPR signal nor the S_2_ state multiline EPR signal, both involving a functional CaMn_4_-cluster, could be detected (EPR spectra not shown).

#### Status of Tyrosine-D oxidation and the quinone acceptors Q_A_ and Q_B_

**Figure 3:**
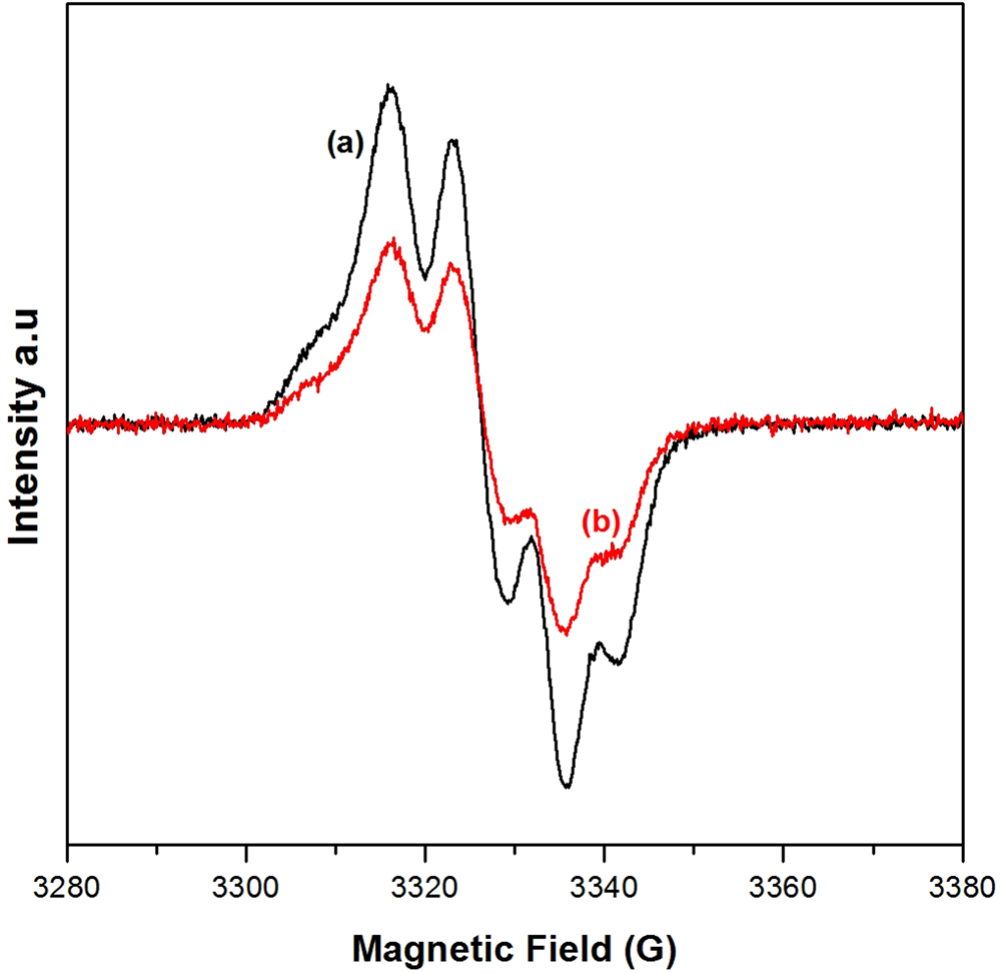
(**a**) The radical EPR spectrum of maximum oxidized Y_D_ (black) as measured from an untreated PS II sample. (**b**) The radical EPR spectrum of oxidized Y_D_ (red) as measured from a similarly concentrated PSII sample where Q_A_ was re-oxidized after double reduction with sodium dithionite/benzyl viologen (see Materials section). EPR conditions: microwave power 1.3 μW, microwave frequency 9.28 GHz, modulation amplitude 3.5 G, temperature 15 K.

Chemical double reduction of Q_A_ is most probably followed by protonation, forming Q_A_H_2_^27–29^. Presumably this species could leave the Q_A_-binding pocket irreversibly during the very long double reduction procedure. However, this does not seem to be the case, at least not in the majority of the PSII centres. In the re-oxidized samples, Y_D_^•^ could be formed in a major fraction of the PS II centres (>55%) (Fig. 3, *red trace*). Y_D_ oxidation involves both primary photochemistry in PSII (charge separation between P_680_ and Pheophytin) and the transfer of, at least, one electron to the quinones on the acceptor side of PS II. Thus the formation of Y_D_^•^ unequivocally shows both that charge separation worked (Figs 2 and 3, *red trace*) and that Q_A_ remained bound in a majority of the samples despite the harsh double reduction/re-oxidation protocols.

**Figure 4:**
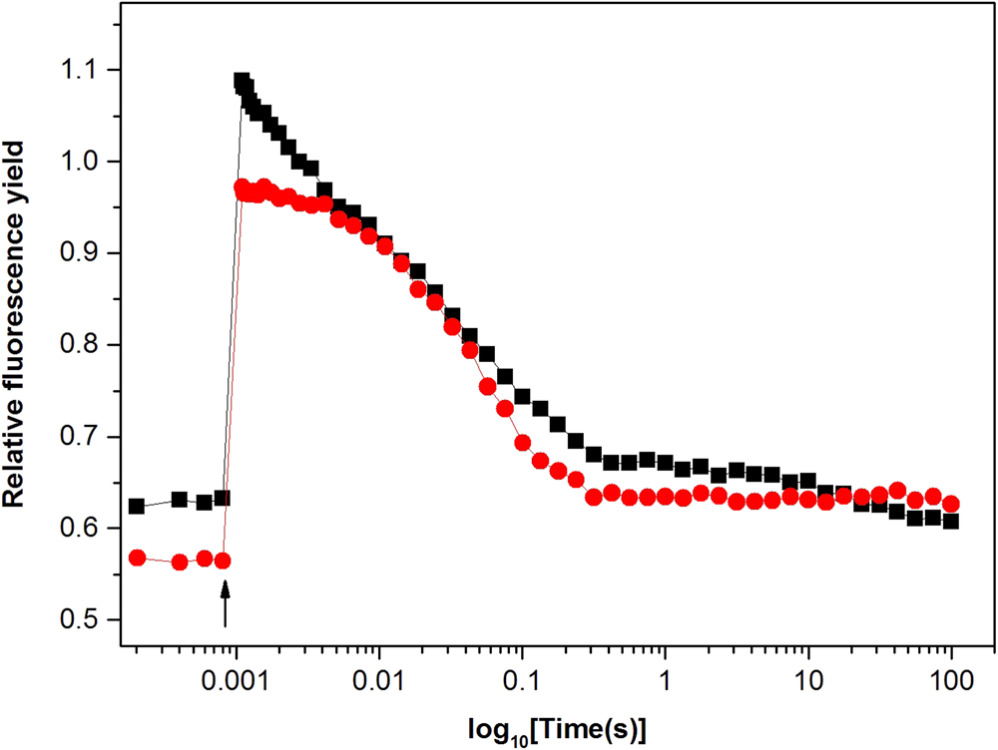
Flash induced fluorescence decay kinetics from the re-oxidized double reduced PS II membrane samples. Traces indicate the fluorescence yield changes after a single flash in the presence (red) or in the absence (black) of DCMU.

We also performed flash induced fluorescence measurements in the re-oxidized samples. Our results show that a large fraction of PSII was able also to perform secondary electron transfer. After the flash, the immediate fluorescence induction is indicative of reduction of Q_A_, when bound to its binding pocket. Q_A_^−^ then decays with different kinetics dependent on the integrity of PSII. The dominating intermediate decay phase in our samples (calculated half-life = 13 ms) (Fig. 4, *black trace*), is similar to the Y_Z_^•^ Q_A_^−^ recombination phase observed in for example Tris-washed PS II membranes^35^. In Tris-washed PSII membranes the OEC is absent, corroborating our conclusion that our samples after double reduction and re-oxidation had lost the CaMn_4_-cluster.

We can also conclude that, not surprisingly, a substantial amount of Q_A_^−^ remained reduced also after the re-oxidation protocol. This is shown by the high *F*_0_ (0.63) in our fluorescence measurement (Fig. 4, *black trace*). Furthermore, there seems to remain very few PS II centres with bound Q_B_ after the re-oxidation procedure. This can be concluded from comparison of the kinetic traces in the presence and absence of the inhibitor DCMU. Addition of DCMU, which inhibits electron transfer from Q_A_ to Q_B_, removed a small fast component from the fluorescence decay curve (Fig. 4, *red trace*). This fast phase most likely reflects electron transfer from Q_A_ to a remaining bound Q_B_. Normally this fast decay phase dominates in PSII and its small amplitude in our samples clearly indicates that there is very little Q_B_ remaining after the double reduction/re-oxidation treatments. The rest of the decay is similar to the decay observed in presence of DCMU and again reflects Y_Z_^•^ Q_A_^−^ recombination.

### Induction of the spin polarized ^3^P680 under white and far-red light excitation

The spin polarized triplet state ^3^P_680_ is a useful probe to the physical environment and photochemistry of P_680_ and the primary radical pair in PSII^36^. In intact PSII, ^3^P_680_ is not observable with EPR spectroscopy^37^. However, when Q_A_ is either removed (in for example the D1/D2/Cyt *b*_***559***_ preparation)^37,38^ or double reduced either by chemical treatment^27–29^ or extensive illumination under anaerobic conditions^39^, the spin polarized EPR signal from ^3^P_680_, can be observed as a result of recombination of the P680^+^ Pheo^−^ charge pair.

**Figure 5:**
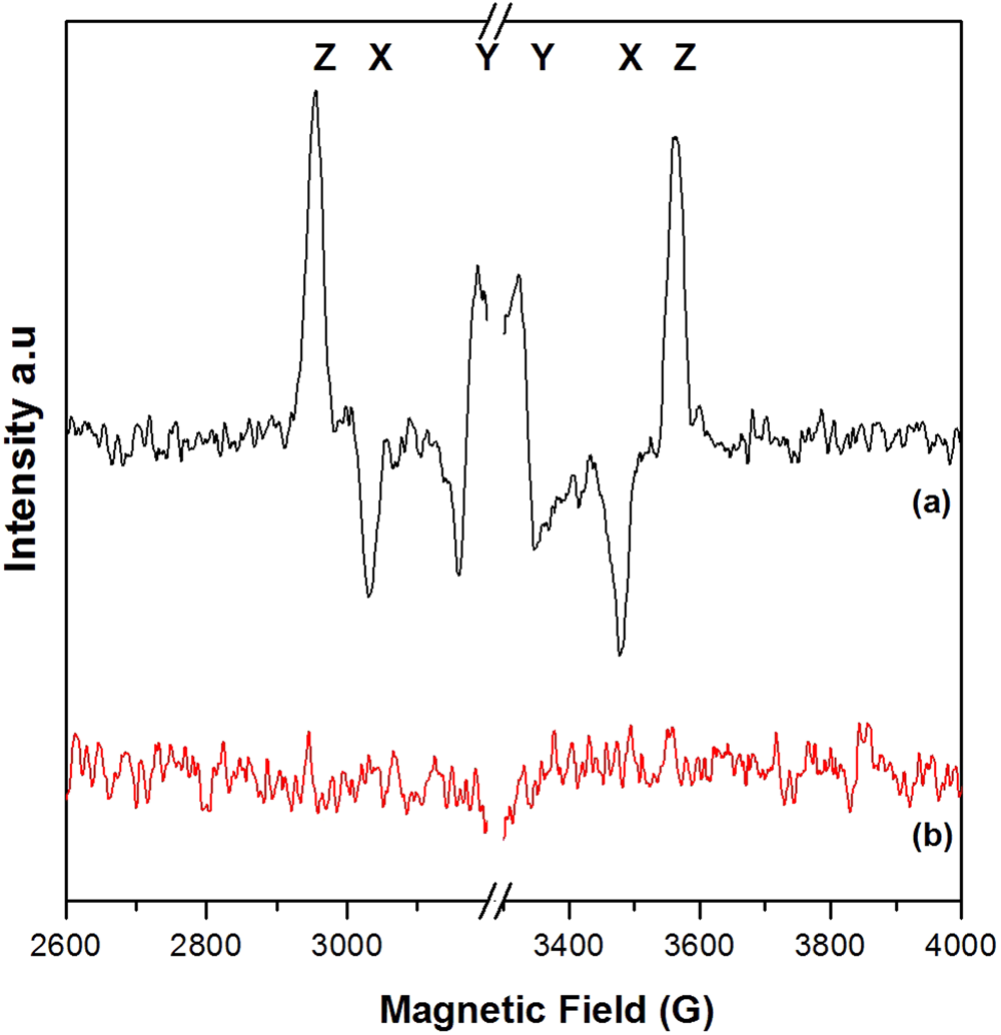
Light minus dark difference EPR spectra showing the formation (or not) of the spin-polarized ^3^P_680_ in PS II samples after reduction with sodium dithionite and benzyl viologen. The light spectra were acquired under continuous illumination at 5 K with (**a**) white light and (**b**) far-red light (732 nm) EPR conditions: microwave power 63 μW, microwave frequency 9.139 GHz, modulation amplitude 30 G, temperature, 5 K.

Here, we have investigated the formation of ^3^P_680_ in samples where Q_A_ has been chemically double reduced. Figure 5 depicts the “*light minus dark”* difference EPR spectra from the spin-polarized triplet state of ^3^P_680_ obtained from PSII membranes with double reduced Q_A_, when illuminated by continuous white light (*spectrum* (a)) at 5 K. Our chemical reduction procedure converts Q_A_ to Q_A_^2−^, which is then protonated to form Q_A_H_2_^27,29^. After excitation with light, no forward electron transfer from Pheo^−^ is possible. Instead, the charge-separated state P_680_^+^ Pheo^−^ will decay through recombination. However, it is long-lived enough to allow spin dephasing, thereby allowing formation of the triplet form of the radical pair, the so called spin-polarized ^3^P_680_ state as follows^36,40^:

$${{\rm{P}}}_{{\rm{680}}}{\rm{Pheo}}\iff {{{\rm{P}}}_{{\rm{680}}}}^{\ast }{\rm{Pheo}}\iff [{{{\rm{P}}}_{{\rm{680}}}}^{+}{{\rm{Pheo}}}^{-}]{\rm{Spin}}\,{\rm{dephasing}}\,\& \,{\rm{recombination}}{\Rightarrow }^{{\rm{3}}}[{{\rm{P}}}_{{\rm{680}}}]$$

Hence, at low temperatures (5 K), the triplet state of the primary donor, ^3^P_680_, is formed with high yield^27,29,38,41^.

From orientation studies of the ^3^P_680_ EPR signal it was concluded that it is localized on a Chl molecule (unidentified at that time) most likely to be Chl_D1_ (in the present structural nomenclature, see Fig. 1)^38,42,43^. It is known that the product of the primary charge separation in visible light is P_D1_^+^ Pheo^−^. Therefore, the localization of the ^3^P_680_ on another Chl molecule than P_D1_ is unexpected. The phenomenon has been explained by two alternative mechanisms. Either the triplet state migrates from P_D1_ to Chl_D1_ after its formation or it is the Chl cation (P_D1_^+^) that moves to Chl_D1_ (forming Chl_D1_^+^) prior to ^3^P_680_ formation (^38^ and refs. therein).

As shown in Fig. 5, *spectrum* (a), the characteristic spin-polarized EPR signal from ^3^P_680_ was observed under white light illumination. The spectrum is in agreement with previously published EPR spectra of ^3^P_680_^27,29,36–38^. The triplet EPR spectrum depicts a low-field to high-field AEEAAE (Absorption = A, Emission = E) polarization pattern spanning ~650 G. X, Y and Z (D > 0, E < 0) indicate the field positions of the respective canonical orientations for the zero field splitting tensor of the triplet state.

We have also intensively searched for the formation of ^3^P_680_ in an identical sample after illumination in the EPR cavity with far-red light. Interestingly, as depicted in Fig. 5, *spectrum* (b), no spin polarized EPR signal from ^3^P_680_ could be detected under these conditions. This obvious difference between visible and far-red light excitation is intriguing as our control measurements clearly indicate that: i) our samples were indeed able to form ^3^P_680_ in visible light (Fig. 5, *spectrum a*) and ii) were indeed able to perform charge separation and a multitude of electron transfer reactions on both the donor and the acceptor side of PS II in both visible and far-red light (Figs 2–4).

#### Accumulated Spectra of spin polarized ^3^P_680_

**Figure 6:**
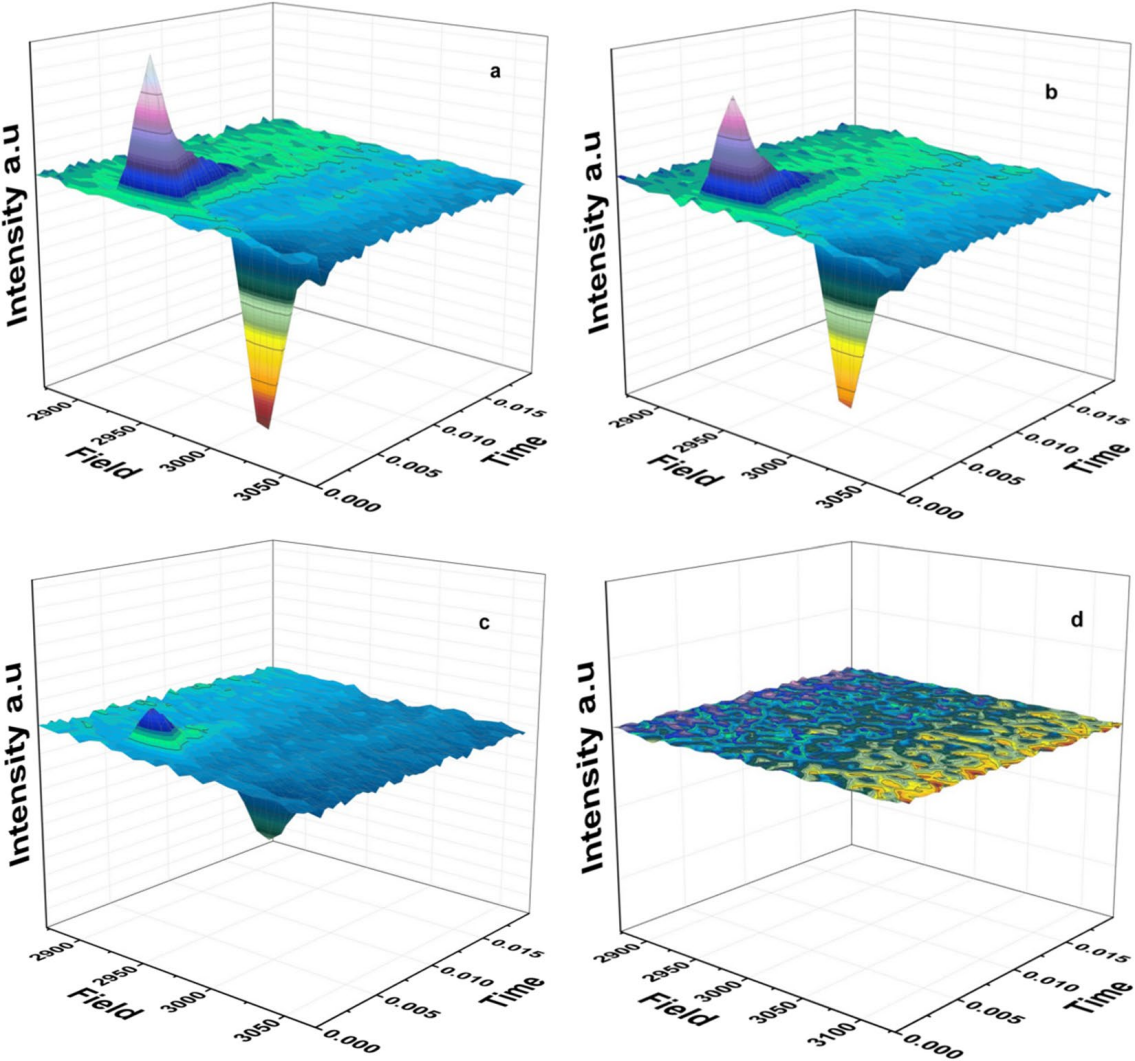
Flash-associated transient 3D spectra of spin polarized ^3^P_680_ at 532 nm (40 mJ) (**a**), 610 nm (35 mJ) (**b**), 689 nm (35 mJ) (**c**) and 730 nm (20 mJ) (**d**) (numbers in parentheses denote the laser flash power), laser flash excitation. Each spectrum represents the accumulation of 1000 transients at 32 field positions (low-field peaks) respectively, with a delay time before flash application of 1 ms, at 5 K. The EPR signal was detected ~50 μs after laser excitation with a detection time window of 20 ms. EPR conditions were as follow: microwave power 63 μW, microwave frequency 9.139 GHz, receiver time constant 0.08 ms, modulation amplitude 35 G, temperature, 5 K.

**Figure 7:**
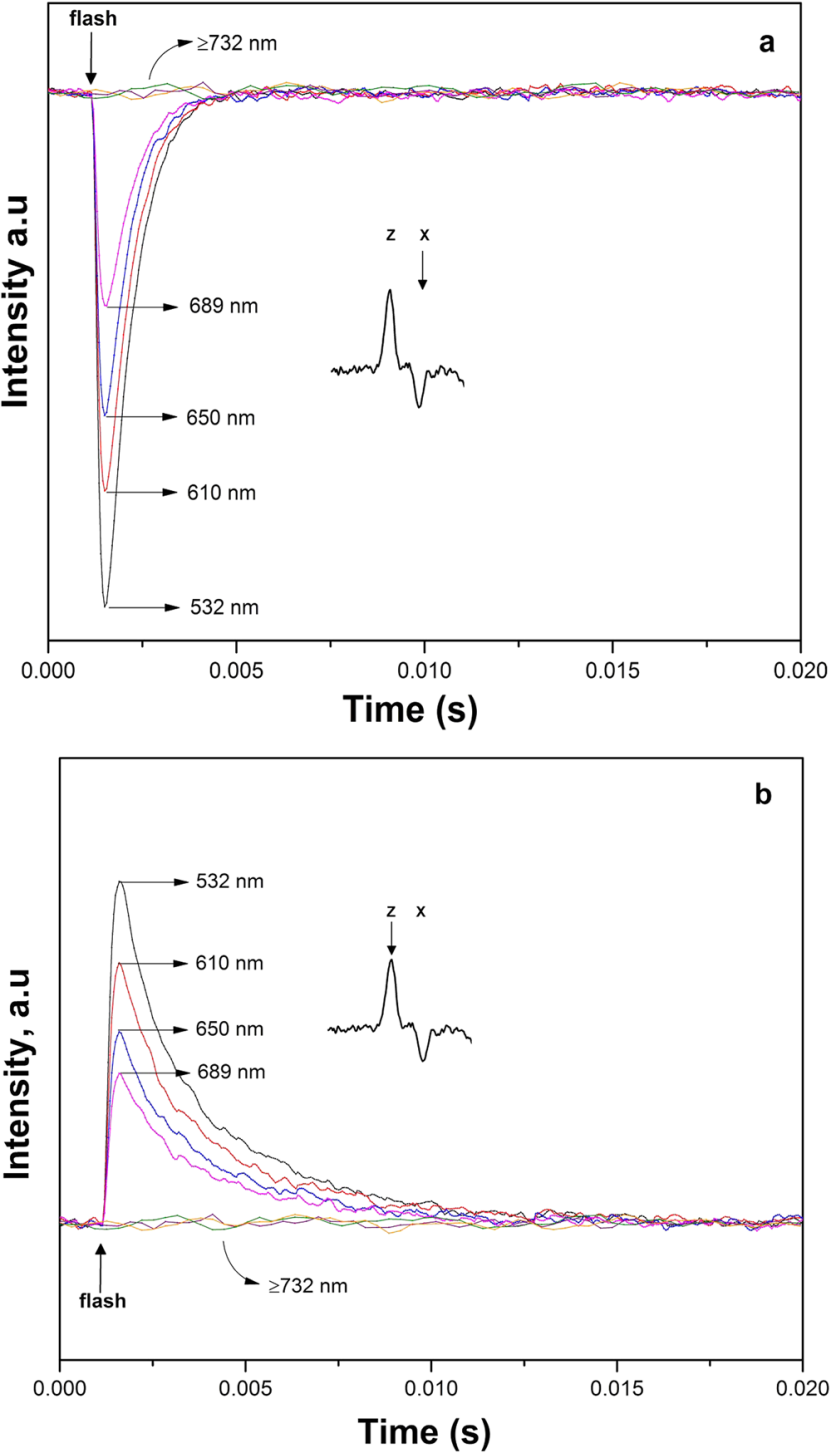
Spin polarized ^3^P_680_ flash-associated transient decay traces upon 532 nm (black), 610 nm (red), 650 nm (blue), 689 nm (pink), 730 nm (purple), 750 nm (orange) and 790 nm (green) flash excitation recorded at fixed field positions X (**a**) and Z (**b**) of the low field region of the ^3^P_680_ EPR spectrum (see inserts and Fig. 4). The traces are the result of 1000 accumulated transients at 5 K. EPR conditions as in Fig. 5.

It is possible that the lack of the ^3^P_680_ EPR signal under far-red light reflects the complete absence of ^3^P_680_ formation. However, it could also be caused by a very fast ^3^P_680_ decaying species not detectable under standard CW EPR conditions. To test this, we therefore accumulated flash—associated spectra at 532 nm, 610 nm, 689 nm, 730 nm, 750 nm and 790 nm at 5 K, recorded with a flash frequency of 5 Hz. Figure 6 shows the flash-associated transients recorded in the low-field region of the ^3^P_680_ spectrum (2850–3150 G) upon 532 nm (a), 610 nm (b), 689 nm (c) and 730 nm (d) laser flash excitation. The intensity of the spin-polarized ^3^P_680_ features is high under 532 nm and 610 nm excitation and decreased by more than 50% under 689 nm excitation. Ultimately, there is no EPR signal from ^3^P_680_ detected even with our fastest available time resolution and after extensive illumination with far-red laser flash excitation (730–790 nm) (Fig. 7). It is also important to point out that the decay kinetics of respectively the X and Z peaks are identical irrespective of the induction wavelength between 532 nm and 689 nm (Fig. 7a,b). The decay kinetics are, however, faster for the X peak than for the Z peak similar to earlier observations^36^. At ≥730 nm no peaks were observed.

## Discussion

The understanding of how low energy excited states contribute to PSII photochemistry continues to be a challenging task^44^. This is especially true if specific charge transfer bands, not detectable by conventional spectroscopy, are responsible for this type of photochemistry^44,45^. In this study we are analysing these phenomena, by using EPR based, comparative product analysis (see also ref.^45^) using the characteristic EPR signal of spin polarized ^3^P_680_ as a probe for the primary charge separation at low temperatures.

In our first publication we showed, for the first time, that PSII operates much further to the far-red region (by 100 nm) than was believed before. We have shown that efficient secondary electron transfer reactions take place under these conditions, including turnover of the *OEC*, reduction of the primary and secondary quinones, oxidation of the secondary donor Tyr_Z_ and reduction of the primary donor, Phe, using far-red (up to ≥800 nm) photons. This was an interesting observation and in an earlier study we proposed an alternative charge separation reaction under far-red conditions involving a low energy primary donor, denoted X^*^, which was able to drive both Pheo^−^ reduction and Y_Z_ oxidation^22^.

This suggestion was further substantiated in a study of low temperature (5 K) electron transfer on the donor side of PS II. To elaborate, we found that far-red light preferentially promoted electron transfer from Y_Z_ to the primary donor. In contrast, visible light promoted almost equal electron transfer from either Y_Z_ or the Cyt *b*_559_/Chl_Z_/Car_D2_ secondary pathway. The partition ratio between the two electron donor pathways was thus wavelength dependent, Y_Z_ being the better donor after far-red excitation^25^.

An important implication of this work, discussed in^22,25^ is that the far-red photochemistry is triggered by weakly absorbing charge transfer states among the core pigments in PSII and not by the well-known bands at 680 nm (P_D1_) or 684 nm (Chl_D1_).

In our present manuscript, we are getting even closer to the primary charge pair, P680^+^ Pheo^−^, showing that the primary donor is indeed different. Thus, our, step wise refined, product analysis technique provides a unique approach to study the far-red photochemistry in PSII and to cast further light on this phenomenon by studying the primary electron donor in PS II, P_680_, directly. Our result is surprising. As described previously, the spin polarized EPR signal from ^3^P_680_ can be observed from PS II centres where Q_A_ has been doubly reduced (or it is absent). It has earlier been shown that its formation using white light is almost quantitative^27,36^. In our hands, the ^3^P_680_ EPR signal is large and fairly consistent in amplitude, when PS II is exposed to light in the visible region. However, in the far-red, at wavelengths ≥730 nm the illumination does not induce any observable ^3^P_680_ EPR signal at all.

There can be several reasons for the above phenomenon. To find the answers and formulate our hypothesis, certain questions must be addressed: (i) Is the electron hole on the primary donor not forming at all under far-red light illumination? (ii) If it was formed in far-red light, would the ^3^P_680_ decay too fast and escape detection? (iii) Do we trigger an alternative charge separation pathway by far-red light, hence creating a different, low energy, state as suggested in ref.^20^? (iv) Is the cation located at a different Chl if compared to the visible light as hypothesized in ref.^23^? If yes, then the recombination between the primary donor and Pheo^−^ occurs too fast to allow ^3^P_680_ formation after far-red illumination.

First, we can exclude that this particular type of PS II sample (sodium dithionite/benzyl viologen reduced) is not able to carry out any charge separation in the far-red at all, since we in fact can photoaccumulate the Pheo^−^ radical in the majority of PS II under illumination with 730 nm light. Therefore, an electron hole on the P_680_ entity is formed after excitation by far-red light. In that case, recombination between Pheo- and P_680_^+^ will take place, either via or without the formation of ^3^P_680_.

Second, in far-red light we observe no spin polarized ^3^P_680_ EPR signal. However, in the event where ^3^P_680_ is anyway formed by the far-red light, it is possible that it decays faster than we can detect it, in contrast to the situation in the visible part of the spectrum. An attempt has been made to investigate this possibility further by our kinetic measurements of ^3^P_680_ induced by different light wavelengths. Here we report identical decay kinetics between the different light regimes where we could observe the ^3^P_680_ in the visible light spectrum. However, we were not able to observe any ^3^P_680_ formation above 700 nm, despite our sub-millisecond time resolution. We argue that there is little reason to suggest that ^3^P_680_ formed by far-red light should decay too fast to allow its detection. Instead, we propose that the far-red light illumination, although it efficiently drives charge separation and creation of a primary radical pair [P_680_^+^ Phe^−^], does not result in the formation of the spin polarized EPR signal from ^3^P_680_. Since Pheo^−^ is the same in both cases, it is the nature of the Chl cation, (Chl^+^) that differs after excitation with visible or far-red light.

This observation also has important implications with respect to the earlier orientation studies of the spin-polarized ^3^P_680_ EPR signal formed by visible light (see above)^38,42,43^. From our results, we can rule out that the triplet state is formed after migration of the cation from one monomeric Chl to another (from P_D1_ to Chl_D1_). In this case we would have observed the ^3^P_680_ EPR signal under both illumination regimes since the triplet would have been formed from the same Chl_D1_^+^ Pheo^−^ charge pair. Instead, detection of the signal under visible light only strongly indicates involvement of different charge pairs under the two illumination regimes with the triplet state only being formed by the P_D1_^+^ Pheo^−^ charge pair recombination.

Third, we have recently demonstrated preferential electron transfer from Y_Z_ in comparison to very weak electron transfer from the Cyt *b*_559_/Chl_Z_/Car_D2_ side donors under far-red light illumination at 5 K^25^. To explain this, we hypothesized the existence of an alternative charge separation pathway under far-red light involving the oxidized primary donor after charge separation residing on Chl_D1_ rather than P_D1_^25^. Thus, we suggest that upon far-red excitation, a new pathway is activated. We denote this as the the Chl_D1_ pathway (Fig. 1). Here, a lower energy excited state [Chl_D1_ Pheo]^*^ is formed, most probably corresponding to the proposed X^*^ in ref.^20^, which would then lead to a different radical pair after the charge separation, [Chl_D1_^+^ Pheo^−^] (Fig. 1).

Lastly, the lack of observable triplet signal implies that the recombination from Pheo^−^ is too fast to allow spin dephasing. This suggests that the electron hole is presumably residing on Chl_D1_ instead of P_D1_. Chl_D1_ is much closer to Pheo than P_D1_ (5 Å vs 8.1 Å)^26^. This shorter distance would probably facilitate faster recombination of the charge pair Chl_D1_^+^ Pheo^−^. Hence, we propose that the far-red light preferentially drives charge separation to Pheo from Chl_D1_ in the reaction center (Fig. 1). Since Chl_D1_^+^ and Pheo_D1_^−^ are in close proximity, we propose that recombination occurs faster than between the radical pair [P_D1_^+^ Pheo^−^] found in visible light. Thus, the [Chl_D1_^+^ Pheo^−^] radical pair probably recombines back to the ground state prior to spin dephasing can occur. Consequently no spin polarized ^3^P_680_ is formed at any of the PSII centres.

It is generally thought that at low temperatures the cation in the primary radical pair is stabilized on P_D1_ (Fig. 1)^40,46–50^, which is not far from neither Y_Z_ nor the Cyt *b*_559_/Chl_Z_/Car_D2_ secondary pathway. Therefore they compete, and both are oxidized in substantial yield at very low temperatures^25^. Contrary, the localization of the primary electron hole on Chl_D1_ rather than P_D1_ under far-red light was discussed in ref.^23^. It was proposed that with the formation of the primary electron hole on Chl_D1_, the location of Chl_D1_ being relatively closer to Y_Z_ but much more distant from the Cyt *b*_559_/Chl_Z_/Car_D2_ secondary pathway, could explain at least qualitatively, the preference for oxidation of Y_Z_ over Cyt *b*_559_/Chl_Z_/Car_D2_.

The existence of parallel charge separation pathways in PSII under different light regimes and under physiological and cryogenic conditions has been suggested and discussed before^50–55^. In 2009 Thapper *et al*.^22^ suggested the formation of a low-energy state denoted X^*^ in PS II after far-red light excitation at room temperature. Although functioning at a lower energetic threshold compared to P_680_^*^, it was shown to effectively trigger the charge separation, reduce Pheo and oxidize Y_Z_ up to ~800 nm. More recently, transient absorption spectroscopy at 77 K, suggested the existence of two different charge separation pathways in PS II^50^. These were dependent on the light induced protein configuration in PSII core complexes. In one path, denoted the Chl_D1_ path, the charge separation events were proposed to involve an interaction between Chl_D1_ and Pheo, and follow the sequence (Chl_D1_ Pheo)* ⇒ (Chl_D1_^+^ Pheo^−^) ⇒ (P_D1_^+^ Pheo^−^). In the second pathway, denoted the P_D1_ path, a charge transfer state P_D1_ P_D2_ is excited and the charge separation events were suggested to follow an alternative sequence (P_D1_ P_D2_ Pheo)* ⇒ (P_D1_^+^ P_D2_ Chl_D1_^−^) ⇒ (P_D1_^+^ P_D2_ Pheo^−^)^52,54,55^. The proposal that the two pathways are actually of an antagonistic nature and the effectiveness of each of the pathways essentially depends on recognition of energetic disorder, fits with our observations.

When a low energy photon in the far-red is used for excitation, the subsequent energetic disorder causes the preferential excitation to be strongly localized at Chl_D1_, thus allowing for charge separation to take place with the first charge separated state being Chl_D1_^+^Pheo^−^. This localized state should be energetically lower than the P_D1_ P_D2_ charge transfer state, which is in agreement with the results of the present study. Thus, we propose that two pathways for primary charge separation exist in parallel in PS II reaction centres and their excitation and activation is wavelength dependent (Fig. 1).

## Concluding Remarks

Here, we have provided further insight into the location of the primary charge separation upon far-red light excitation of PS II, using the EPR signal of the spin polarized ^3^P_680_ as a probe. We demonstrate that, under far-red light illumination, the spin polarized ^3^P_680_ is not formed, although we show that the primary charge separation is still occurring at these conditions. We propose that this is because under far-red light excitation, the primary electron hole is localized on Chl_D1_, rather than on P_D1_ (Fig. 1). The formation of the lower energy excited state Chl_D1_ Pheo^*^ by far-red light, leads to the [Chl_D1_^+^ Pheo^−^] primary charge pair. The close proximity of these two cofactors, allows fast recombination to the ground state before spin dephasing can occur, hence the ^3^P_680_ cannot be formed.

The fact that identical samples have demonstrated charge separation upon both far-red and visible light excitation further supports our hypothesis that two pathways for primary charge separation exist in parallel in PS II reaction centres. They are excited and activated dependent of the wavelength applied. In the visible part of the spectrum the first product of the charge separation is normally considered to be P_D1_^+^ Pheo^−^.

In stark contrast, we hypothesize that far-red illumination results in the Chl_D1_^+^ Pheo^−^ radical pair. The latter recombines without formation of the ^3^P_680_, thereby explaining the results in the present contribution. When far-red light is applied to intact PS II however, the oxidizing electron hole, presumably Chl_D1_^+^, preferentially drives electron transfer from Y_Z_ and consequently the CaMn_4_ cluster. As described earlier, the Yz-CaMn_4_ pathway is preferred over the Cyt *b*_559_/Chl_Z_/Car_D2_ pathway at low temperatures due to the favourable location of Chl_D1_ vs Y_Z_ over Chl_Z_^21^.

## Materials and Methods

### PSII membrane preparation

Spinach (*Spinacia oleracia*) was grown hydroponically as described previously at 20 °C under cool white fluorescent light (Osram Powerstar HQI-400W/DV dysprosium lamp, intensity 300 μEm^−2^s^−1^), with light-dark periods of 12 h^33^. Oxygen evolving PSII enriched membranes (BBY-type) were prepared according to previously published procedures^56,57^. The membrane particles were re-suspended in a final buffer containing 400 mM Sucrose, 15 mM NaCl, 3 mM MgCl_2_ and 25 mM MES-NaOH pH 6.1, and frozen as beads at −80 °C, at a Chl concentration of 6 mg/ml.

### Chemical reduction and re-oxidation of PS II

In order to obtain reaction centers with doubly reduced Q_A_, PS II membranes were exposed to reducing conditions as described in^27,28^ with modifications as in^29^. Specifically, upon addition of 40 mM sodium dithionite, 100 μM benzyl viologen and 3 mM EDTA under anaerobic conditions, dark-adapted PS II membranes at a Chl concentration of 6 mg/ml, were incubated in the dark for 5 hrs to achieve the double reduction of Q_A_ to Q_A_H_2_. All incubations were carried out in argon flushed EPR tubes. Upon completion of the incubation time, the EPR samples were frozen within 1 sec in a 200 K dry ice/ethanol bath and subsequently transferred into N_2_(l) before the measurements.

“Reversed” samples where Q_A_H_2_ was re-oxidized, were prepared according to previously published procedures^27^. The doubly reduced samples were initially washed three times (15 000 × g, 15 min cycle) with argon flushed buffer containing 400 mM Sucrose, 15 mM NaCl, 3 mM MgCl_2_ and 25 mM MES-NaOH pH 6.1, to remove sodium dithionite and benzyl viologen. They were then re-oxidized with 5 mM K_3_Fe(CN)_6_, which was subsequently removed by repeating the washing procedure three times with final buffer, as described above. All washing steps were performed in complete darkness and at 4 °C.

### Illumination procedures

The spin polarized ^3^P_680_ EPR signal was induced by illumination of the samples in the EPR cavity with white or far-red light at 5 K. For continuous illumination, white light, filtered through a 5 cm-thick copper sulphate solution heat filter, was provided by an 800 W halogen projector lamp. Far-red light illumination was achieved by the use of a custom made LED module, emitting continuous light centred at 732 nm. Appropriate long pass filters were used so as to ensure blocking of transmission of any stray UV light (CC4) as well as transmission of light <725 nm (RG9). Transient formation and accumulation of the spin polarized ^3^P_680_ EPR signal was studied by applying laser flashes into the EPR cavity, from a Spectra Physics PRO-290 Q-switched Nd:YAG laser (6 ns flash, 5 Hz flash frequency) equipped with a Spectra Physics Quanta Ray MOPO 730 optical parametric oscillator.

The Split S_1_ signal was obtained by white or far-red continuous wave light illumination at 5 K, as in^58^.

For the measurements of the Pheo^−^ radical, doubly reduced PS II membranes (6.0 mg Chl/ml) were subjected to white or far-red (732 nm) continuous wave light illumination as described earlier^30–32^, at 20 °C, for 6 and 10 minutes respectively at room temperature. Thereafter, the samples were frozen within 1 sec in a 200 K dry ice/ethanol bath and subsequently transferred into N_2_(l) before the measurements. The size of the EPR spectrum of the formed Pheo^−^ radical was compared by double integration to the size of the EPR spectrum from Y_D_^•^ in a corresponding sample prior to double reduction, allowing us to quantify the function of the primary charge separation in the double reduced samples.

### Fluorescence measurements

Flash-induced fluorescence decay measurements were performed at room temperature at a sample concentration of 20 µg Chl/ml. PS II membranes were dark adapted for 5 min and measurements were performed in the presence or absence of 20 µM DCMU. The variable fluorescence decay traces were recorded with a FL3000 double modulated fluorometer (PSI Photon Systems Instruments, Czech Republic) according to^59^. The first data point was taken 100 μs after the actinic flash. Measuring flashes were then applied logarithmically eight times per decade in a time range up to 100 s^60^.

### EPR spectroscopy

X-band EPR measurements were performed with an Elexsys E580 (Bruker BiosSpin) equipped with a standard cavity (ST 4102). All measurements were performed at low temperature that was achieved with the use of a helium flow cryostat and an ICT-4 temperature controller (Oxford Instruments, UK). The time-resolved EPR (TREPR) measurements were performed with the use of the ADF fast digitizer board (2 MHz fixed sampling rate). Signal acquisition was synchronized with 5 Hz laser flashes using a LC880 TTL pulse generator (100 MHz internal clock) (LabSmith, Livermore, California). An accessory transformer was used to amplify the TTL pulses to the 5 V amplitude required for triggering the laser lamp and Q-switch. Analysis of the EPR spectra was carried out with the Bruker Xepr 2.1 software.
